# Medical Significance of Uterine Corpus Endometrial Carcinoma Patients Infected With SARS-CoV-2 and Pharmacological Characteristics of Plumbagin

**DOI:** 10.3389/fendo.2021.714909

**Published:** 2021-10-12

**Authors:** Yongming Li, Songzuo Yu, Yu Li, Xiao Liang, Min Su, Rong Li

**Affiliations:** ^1^ Department of Gynecology, Guigang Maternal and Child Health Care Hospital, Guigang, China; ^2^ Department of Neurosurgery, Guigang City People’s Hospital, The Eighth Affiliated Hospital of Guangxi Medical University, Guigang, China; ^3^ Laboratory of Environmental Pollution and Integrative Omics, Guilin Medical University, Guilin, China; ^4^ Guangxi Key Laboratory of Tumor Immunology and Microenvironmental Regulation, Guilin Medical University, Guilin, China

**Keywords:** cancer, clinical, pharmacologic (drug) therapy, target, mechanism and characterization

## Abstract

**Background:**

Clinically, evidence shows that uterine corpus endometrial carcinoma (UCEC) patients infected with severe acute respiratory syndrome coronavirus 2 (SARS-CoV-2) may have a higher death-rate. However, current anti-UCEC/coronavirus disease 2019 (COVID-19) treatment is lacking. Plumbagin (PLB), a pharmacologically active alkaloid, is an emerging anti-cancer inhibitor. Accordingly, the current report was designed to identify and characterize the anti-UCEC function and mechanism of PLB in the treatment of patients infected with SARS-CoV-2 *via* integrated *in silico* analysis.

**Methods:**

The clinical analyses of UCEC and COVID-19 in patients were conducted using online-accessible tools. Meanwhile, *in silico* methods including network pharmacology and biological molecular docking aimed to screen and characterize the anti-UCEC/COVID-19 functions, bio targets, and mechanisms of the action of PLB.

**Results:**

The bioinformatics data uncovered the clinical characteristics of UCEC patients infected with SARS-CoV-2, including specific genes, health risk, survival rate, and prognostic index. Network pharmacology findings disclosed that PLB-exerted anti-UCEC/COVID-19 effects were achieved through anti-proliferation, inducing cytotoxicity and apoptosis, anti-inflammation, immunomodulation, and modulation of some of the key molecular pathways associated with anti-inflammatory and immunomodulating actions. Following molecular docking analysis, *in silico* investigation helped identify the anti-UCEC/COVID-19 pharmacological bio targets of PLB, including mitogen-activated protein kinase 3 (MAPK3), tumor necrosis factor (TNF), and urokinase-type plasminogen activator (PLAU).

**Conclusions:**

Based on the present bioinformatic and *in silico* findings, the clinical characterization of UCEC/COVID-19 patients was revealed. The candidate, core bio targets, and molecular pathways of PLB action in the potential treatment of UCEC/COVID-19 were identified accordingly.

## Highlights

- Clinical characteristics of UCEC/COVID-19 patients, including specific genes, health risk, survival rate, and prognosis, were determined.
- All candidates, core targets of PLB in treatment for UCEC/COVID-19, were screened out and identified.
- Biological functions and molecular pathways of PLB in treatment for UCEC/COVID-19 were revealed.
- Biological docking findings elucidated that PLB mediated effective molecular affinity with COVID-19.

## Introduction

Coronavirus disease, caused by SARS-CoV-2, is continuously evolving and spreading around the world in 2020, especially in India (1). In western countries, including the United States, increasing reports indicate that the disease incidence, death rate, and infection rate of COVID-19 are elevating sharply owing to limited management (2). While the vaccine against coronavirus is still developing, there is still no clinical medicine to treat COVID-19 because of the current evolving situation (3). In addition to adjuvant therapy, some common drugs, such as hydrocortisone, may alternatively be prescribed to patients infected with SARS-CoV-2; however, the clinical effectiveness against COVID-19 remains limited (4). Taken together, mankind needs to screen and develop some pharmacologically bioactive compounds for the treatment of COVID-19, an emerging and evolving epidemic. In clinical practice, it has been reported that hospitalized patients with cancer seem to be highly susceptible to infection with coronavirus, resulting in an additional death toll (5). Uterine corpus endometrial carcinoma (UCEC) is a gynecological cancer characterized by biological invasiveness and potent metastasis (6). Epidemiologically, it is estimated that the incidence and mortality of UCEC are increasing in recent decades, especially in less-resourced nations, and it is still a major public health problem worldwide (7). In China, other reporting data show that the death rate of young women with UCEC is increasing yearly, especially in urban areas (8). Accordingly, cancer patients, who may be hospitalized during clinical treatment, may have a higher risk of exposure to SARS-CoV-2 infection during the early stages of COVID-19 as the novel coronavirus is transmitting rapidly, and it is not detectable during an outbreak (9). Theoretically, it could be reasoned that some of the UCEC patients that were hospitalized appear to have a high risk of infection with SARS-CoV-2. Thus, the treatment of UCEC/COVID-19 patients will become more challenging as there are no current medicines to treat UCEC and COVID-19, and the virus is still evolving in the world. Accordingly, anti-UCEC/COVID-19 medicine is urgent to be screened and developed in the current situation.

Plumbagin (PLB), a bioactive naphthoquinone, exerts potent pharmacological properties against some of the chronic diseases, such as obesity and fatty liver (10). In particular, PLB functions as an anti-cancer compound inducing cytotoxicity and suppressing cancer cells (11). The preliminary anti-tumor mechanism exerted by PLB would be through targeting the Wnt/β-catenin and AKT signaling pathways (12, 13). Interestingly, our previous findings suggest that PLB plays a potent anti-cancer effect against pancreatic cancer and hepatocellular carcinoma (14, 15). Furthermore, preclinical data indicate that PLB exerts antiproliferative activity against cervical cancer cells (16, 17). However, it has been reported that PLB has effective anti-inflammatory effects *via* the suppression of nuclear factor-κB activity (18). Moreover, it is also reported that PLB can possess antibacterial effects, including action against *Staphylococcus aureus* and *Bacillus subtilis* (19, 20). However, the potential relevance of PLB in UCEC has not been assessed. Moreover, the anti-UCEC/COVID-19 functions and mechanisms achieved by PLB remain uninvestigated. The evolving methodology of *in silico* analysis, which includes network pharmacology and molecular docking analysis, can be used for unmasking the functions and mechanism of phytocompounds to treat a medical disease before clinical investigation (21, 22). The present study was designed to determine the clinical characteristics of UCEC/COVID-19, to find an anti-UCEC/COVID-19 bio target, and to determine the mechanism exerted by PLB through network pharmacology and biological molecular docking analysis.

## Materials and Methods

### Selection of UCEC/COVID-19-Functional Genes

In order to select and define UCEC/COVID-19-functional genes, we used the Cancer Genome Atlas (TCGA) portal (https://portal.gdc.cancer.gov/) to download the data of Gene Expression Quantification (GEQ) in UCEC’s transcriptome profiling, accessed on September 14, 2020. Using Bioconductor’s “limma” package in *R*-language to screen GEQ, the criteria were set as false discovery rate (FDR) and |log^fold change^| > 1 for obtaining the differential genes of UCEC. In addition, we used different gene-functional modules from the Genecard database, Online Mendelian Inheritance in Man (OMIM) database, Therapeutic Target Database (TTD), and National Center for Biotechnology Information (NCBI). Next, the differential genes of UCEC and COVID-19 targets were subjected to a map through the online bioinformatic Venn diagram tool, in order to identify all the UCEC/COVID-19 shared targets (22, 23).

### Clinical Information Determination of UCEC/COVID-19-Functional Genes

After processing the harvested clinical data downloaded from the TCGA database, the UCEC/COVID-19-functional genes were obtained similarly. The survival prognosis for a UCEC/COVID-19 case was assessed using the “survival” package in R-language. The prognostic determination was conducted through univariate Cox proportional hazards regression analysis. Meanwhile, the clinical characterization of UCEC/COVID-19 in patients was determined with the multivariate Cox proportional hazards regression model. Following the average risk score, patients were grouped into low and high-risk populations (24, 25).

### Analysis of Candidate and Shared Genes in UCSC/COVID-19

We identified and harvested the functional genes of PLB through different online platforms: Swiss Target Prediction, Batman, Genecards, and SuperPred webserver. After gene correction, the candidate genes of PLB and UCSC/COVID-19 were further mapped using the online Venn diagram to harvest all intersection targets (26, 27).

### Gene Ontology and Signaling Pathway Enrichment Analyses of Intersection Targets

We used R-language package settings, including “ClusterProfiler,” “ReactomePA,” “org.Hs.eg.Db,” and “GOplot,” to conduct the gene analysis with all intersection targets, as reported elsewhere (28). Next, to analyze the biological processes, the Kyoto Encyclopedia of Genes and Genomes (KEGG) pathway enrichment was determined and visualized. Gene annotation information harvested from “org.Hs.eg.Db”, p-value Cutoff = 0.05, q-value Cutoff = 0.05 during enrichment, contributing to output the corresponding histogram.

### Screening Core Targets of Plumbagin in the Treatment of COVID-19/UCEC

All interaction targets of PLB and COVID-19/UCEC were loaded into the STRING tool (version 11.0) for functional protein association networks. Then the network interaction relationship between the target and target function-related proteins was obtained. Accordingly, target interaction in the PPI network diagram and the tsv data were produced and collected. We applied the NetworkAnalyzer in Cytoscape_v3.7.1 to analyze the topological parameters, such as the median and maximum degrees of freedom in the network. All core targets were screened according to degree value, and the upper limit of the filtering range was the maximum degree value in the topology data, whereas the lower limit was the median degree of freedom (29).

### Construction of Network-Connected Visualization

Further, we used Cytoscape_v3.7.1 to construct a drug-target gene ontology biological process pathway disease visualization graph based on the results of PLB intervention in COVID-19/UCEC’s GO-based biological process and pathway enrichment, as described previously (30).

### Molecular Docking Assessment

Further, we used molecular docking determination to predict and identify the binding capacity and interaction force between associated proteins, including MAPK3, TNF, PLAU, and the PLB molecule. Accordingly, the compound structures of PLB and Darunavir were obtained from PubChem database (https://pubchem.ncbi.nlm.nih.gov/), and all investigated proteins for docking were gained from Protein Data Bank database (https://www.rcsb.org/). The PLB compound structure was optimized the minimum energy 2 (MM2) through 3D Draw module in the Chem Bio Office software. Ligand-associated PDBQT structure file format was necessary file for virtual screening. The PDBQT file of PLB compound structure was generated by using Raccoon software. And the investigated proteins/targets were processed through MGLTools (1.5.6 version), a supporting tool of Autodock Vina software, followed with hydrogenation, Gasteiger charge calculation, non-polar hydrogen combination. The original pdb file format was converted to recognized format by the Autodock Vina setting, providing ligand basis for chemically and structurally docking. Docking active center, including surrounding residues centered on the original ligand, was set by using Grid box function setting. The rationality of docking parameter settings in PLB and proteins/targets were determined according to the size of root mean square deviation (RMSD) between the docked and original ligand molecules. It was generally reasoned that RMSD ≤ 4 Å was the threshold for the conformation of ligand to match the original ligand docked. More details of the procedures were reported previously (31, 32). Additionally, two-dimensional diagrams of PLB and Darunavir with known 3D structure were created according to chemical drawing conventions in PoseView tool. And generation of structure diagrams and layout modifications were plotted using the library 2Ddraw tool.

## Results

### Identifying Candidate Genes of COVID-19 and UCEC

**Figure 1:**
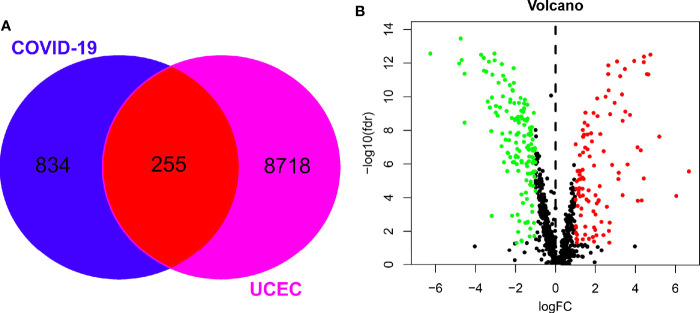
Screening of mutual genes between UCEC and COVID-19. **(A)**, candidate, mutual genes of UCEC and COVID-19 in a Venn diagram. **(B)**, differentially expressed genes from mutual genes shown in a volcano-plot map.

Using the Genecard and OMIM online tools, we identified a group of 1089 genes of COVID-19. A set of 8973 associated UCEC genes were determined using the TCGA database. Following the online Venn diagram assay, we collected a total of 255 mutual genes of COVID-19 and UCEC (**Figure 1A**). Finally, the volcano map of the mutual genes was obtained by R-language analysis, in which 108 genes were up-regulated and 147 others were down-regulated (**Figure 1B**).

### Clinical and Medical Characteristics in COVID-19/UCEC

**Figure 2:**
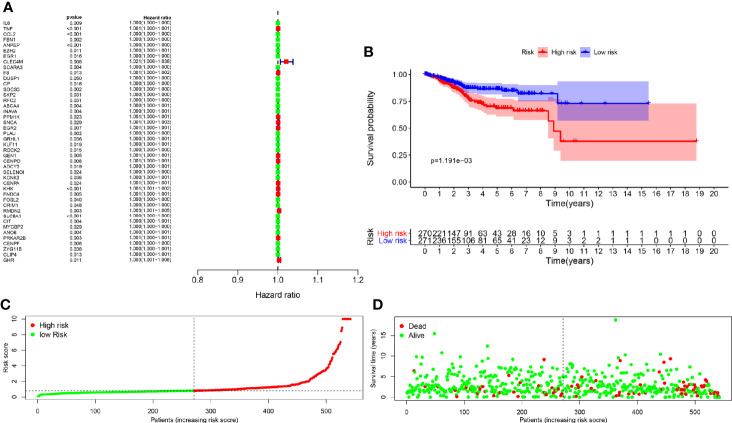
Clinical features of UCEC/COVID-19-related genes. **(A)**, Univariate Cox determination was conducted to identify 44 candidate genes. **(B)**, Multivariate Cox analysis identified 13 specific genes, including CCL2, ANPEP, CLEC4M, SCARA3, CP, ABCA4, KHK, SLC8A1, ZYG11B, GHR, TNF, FOSL2, and PLAU. The survival analysis suggested statistical significance associated with 13 specific genes in the overall survival between high- and low-risk groups. **(C, D)**, Further analysis showed the greater the patient’s risk value, the higher the patient’s risk score; similarly, higher mortality and lower survival.

**Figure 3:**
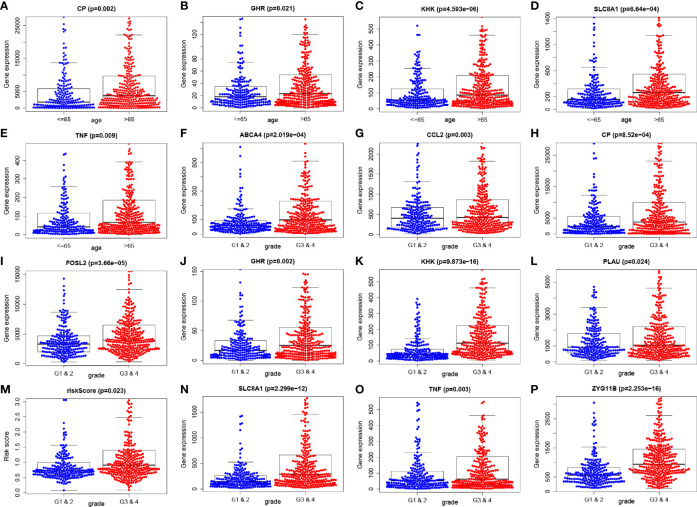
Clinical correlation analysis of 13 genes was carried out and assessed; the results showed that each gene has no correlation with clinical single factors **(A–P)**.

There were 44 genes with P < 0.05 in the univariate Cox analysis among the 255 differentially expressed genes, as detailed in **Figure 2A** and **Table 1**. Further, we screened out the other 13 genes from the 44 specific genes through multivariate Cox analysis performed with hazard regression analysis, including CCL2, ANPEP, CLEC4M, SCARA3, CP, ABCA4, KHK, SLC8A1, ZYG11B, GHR, TNF, FOSL2, and PLAU (**Table 2**). Based on the logistic regression coefficient (coef) value for patient risk evaluation, the risk value was the sum of the expression of each gene multiplied by the coef value, thereby dividing patients into high-risk and low-risk groups. As shown in the survival analysis, we found that the high- and low-risk groups related to 13 genes had a statistical significance on overall survival (**Figure 2B**); the greater the patient’s risk value, the higher the patient’s risk score (**Figure 2C**). Likewise, a higher mortality and lower overall survival were observed (**Figure 2D**). We also carried out single factor and multivariate independent prognostic analyses of the 13 associated genes. As a result, age factor had a significant difference in the single factor analysis P < 0.05, while grade and risk value had significant differences in both single factor and multivariate independent prognostic analyses. Similarly, the hazard ratio was greater than 1. The result showed the higher the risk value, the greater the prognostic risk, and it can be used as an independent prognostic analysis factor for COVID-19/UCEC (**Table 3**). Clinical correlation analysis of these 13 genes was further performed, and the results showed that each gene had no correlation with clinical single factors, as detailed in **Table 4** and **Figure 3**.

**Table 1:** Univariate cox proportional hazards regression analysis of COVID-19_UCEC gene.

Genes	HR	HR.95L	HR.95H	p-value
IL6	1.000207554	1.000052316	1.000362816	0.008778943
TNF	1.000753076	1.000353788	1.001152523	0.000217956
CCL2	1.000273964	1.000143442	1.000404503	3.88E-05
FBN1	1.000213328	1.000078541	1.000348132	0.001921055
ANPEP	1.000020478	1.000009404	1.000031554	0.000289922
EZH2	1.000291755	1.000066441	1.00051712	0.011148452
EGR1	1.000024347	1.000004519	1.000044175	0.016100539
CLEC4M	1.021467315	1.005536209	1.037650824	0.008088622
SCARA3	1.000071903	1.000022389	1.00012142	0.004423996
F8	1.001312422	1.000281938	1.002343967	0.012540539
DUSP1	1.000042068	1.000000058	1.00008408	0.049682011
CP	1.00000685	1.000001293	1.000012407	0.015698793
SOCS3	1.000066755	1.000023813	1.000109699	0.002312255
SKP2	1.000236732	1.000021935	1.000451576	0.030762207
RFC2	1.000238896	1.000022052	1.000455787	0.03082664
ABCA4	1.000393186	1.000123976	1.000662468	0.004199901
INAVA	1.000351216	1.000111279	1.00059121	0.004116141
PPM1K	1.00050373	1.000069526	1.000938122	0.022973265
SNCA	1.001338861	1.000133987	1.002545187	0.029401172
EGR2	1.000738441	1.000204013	1.001273155	0.00676005
PLAU	1.000078444	1.000029525	1.000127365	0.001672441
GRHL1	1.000423737	1.000027483	1.000820148	0.036088497
KLF11	1.000478342	1.000078569	1.000878274	0.019013985
ROCK2	1.00024856	1.000047953	1.000449207	0.015159967
GEN1	1.000503887	1.000155033	1.000852863	0.004637324
CENPO	1.000660487	1.000186113	1.001135086	0.006349271
ADCY3	1.000405922	1.000066913	1.000745045	0.018930544
SELENOI	1.000198502	1.000026436	1.000370597	0.023751878
KCNK3	1.000291018	1.000019436	1.000562674	0.0357067
CENPA	1.000547601	1.000071496	1.001023932	0.024172924
KHK	1.001353007	1.000581099	1.002125511	0.000589413
FNDC4	1.000803206	1.00024437	1.001362354	0.004841882
FOSL2	1.000052958	1.000002379	1.000103539	0.040155414
CRIM1	1.000193521	1.00000154	1.000385539	0.048189646
RMDN2	1.003267988	1.001117153	1.005423445	0.002885881
SLC8A1	1.000303821	1.000142034	1.000465635	0.000232414
CIT	1.000309325	1.000099374	1.00051932	0.003879488
MYCBP2	1.000211944	1.000021642	1.000402281	0.029044014
ANO6	1.000306132	1.000100554	1.000511752	0.003513997
PRKAR2B	1.00051216	1.000170903	1.000853534	0.00326338
CENPF	1.000063814	1.000018577	1.000109054	0.005694564
ZYG11B	1.000328864	1.00001874	1.000639084	0.037670898
CLIP4	1.000466365	1.000099814	1.000833051	0.012638528
GHR	1.00338401	1.000762567	1.006012319	0.011371407

**Table 2:** Multivariate ox proportional hazards regression analysis.

Genes	coef	HR	HR.95L	HR.95H	p-value
CCL2	0.000307056	1.000307103	1.000155931	1.000458298	6.84E-05
ANPEP	2.78E-05	1.000027785	1.000015648	1.000039923	7.22E-06
CLEC4M	0.01810549	1.018270388	1.000379462	1.036481279	0.045294101
SCARA3	6.89E-05	1.000068931	1.000013251	1.000124614	0.015249326
CP	1.10E-05	1.000011016	1.000001055	1.000020976	0.030194505
ABCA4	0.000426783	1.000426874	1.000059006	1.000794878	0.02294149
KHK	0.001078914	1.001079496	1.00003703	1.002123048	0.042394865
SLC8A1	0.000344485	1.000344545	1.000107186	1.000581959	0.004438439
ZYG11B	-0.000579096	0.999421071	0.998842689	0.999999788	0.049916327
GHR	0.003522729	1.003528941	1.000308451	1.0067598	0.031712525
TNF	0.000456603	1.000456708	0.999959582	1.000954081	0.071769993
FOSL2	-7.33E-05	0.999926709	0.999851297	1.000002126	0.056816556
PLAU	6.46E-05	1.000064581	0.999997251	1.000131916	0.060117016

**Table 3:** Univariate and multivariate analysis of the correlation of 13 gene expression values with OS among the patients.

Parameter	Univariate analysis			Multivariate analysis		
	HR	95% CI	p-value	HR	95% CI	p-value
age	1.0323	1.0100-1.055	4.32E-03	1.0166	0.9946-1.0391	1.41E-01
Grade (G1-G4)	2.5350	1.7539-3.6639	7.45E-07	2.5078	1.7553-3.5830	4.40E-07
riskScore	1.1632	1.1275-1.2000	1.98E-21	1.1743	1.1318-1.2184	1.23E-17

**Table 4:** Clinical correlation analysis.

Genes	Age (≤65 *vs* >65)	Grade (G1 & 2 *vs* G3 & 4)
CCL2	0.681 (0.496)	-2.983 (0.003)
ANPEP	-1.442 (0.150)	0.992 (0.322)
CLEC4M	-1.444 (0.149)	-0.509 (0.611)
SCARA3	0.474 (0.636)	-0.799 (0.425)
CP	-3.088 (0.002)	-3.358 (8.52e-04)
ABCA4	0.437 (0.662)	-3.757 (2.019e-04)
KHK	-4.633 (4.593e-06)	-8.329 (9.873e-16)
SLC8A1	-3.424 (6.64e-04)	-7.242 (2.299e-12)
ZYG11B	-1.934 (0.054)	-8.476 (2.253e-16)
GHR	-2.31 (0.021)	-3.085 (0.002)
TNF	-2.605 (0.009)	-2.976 (0.003)
FOSL2	-0.29 (0.772)	-4.163 (3.66e-05)
PLAU	0.169 (0.866)	-2.261 (0.024)
riskScore	-0.617 (0.537)	-2.277 (0.023)

### Identification of Candidate and Mutual Targets of PLB and COVID-19/UCEC

**Figure 4:**
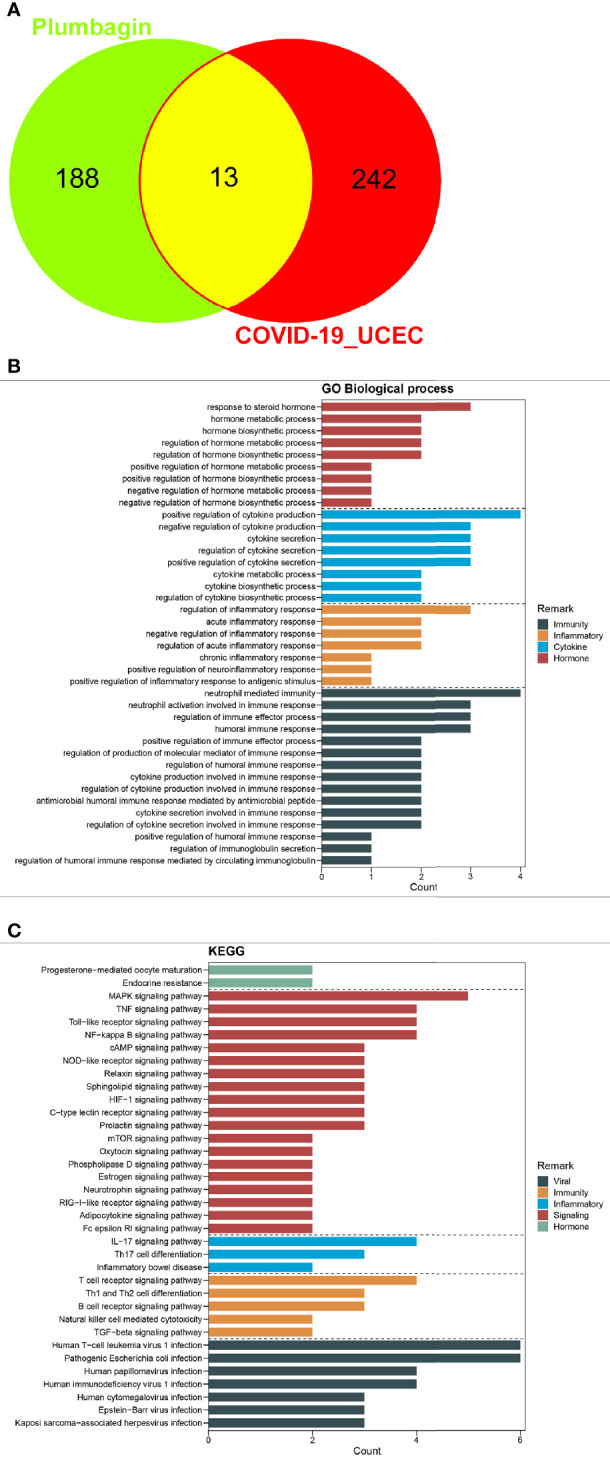
Preliminary findings of network pharmacology. **(A)**, The candidate, mutual genes of PLB and UCEC/COVID-19 in a Venn diagram. **(B, C)**, GO-based biologic process and KEGG-based signaling pathway of PLB against UCEC/COVID-19 following enrichment analysis.

Following with the Swiss Target Prediction, TCMSP databases for screening drug targets, a series of 201 PLB-pharmacological genes were identified after correcting by the Uniprot database and removing duplicates. As a result, the 13 mutual genes of PLB and COVID-19/UCEC were screened out through an online bioinformatics platform (**Figure 4A**; more details shown in **Supplemental Table 1**).

### Enrichment Outcomes of Gene Ontology and Molecular Pathway

All mutual genes were further used for GO and KEGG enrichment assays. The findings indicated the detailed GO-assayed biological processes (**Figure 4B**) and KEGG signaling pathways (**Figure 4C**) of PLB in the treatment of COVID-19/UCEC. The biological processes were mainly the regulation of cytokine secretion involved in immune response, cytokine secretion involved in immune response, neutrophil-mediated immunity, antimicrobial humoral immune response mediated by antimicrobial peptide, regulation of cytokine production involved in immune response, humoral immune response, cytokine production involved in immune response, regulation of immune effector process, regulation of humoral immune response, regulation of production of molecular mediator of immune response, neutrophil activation involved in immune response, regulation of humoral immune response mediated by circulating immunoglobulin, positive regulation of immune effector process, regulation of immunoglobulin secretion, positive regulation of humoral immune response, regulation of inflammatory response, regulation of acute inflammatory response, negative regulation of inflammatory response, positive regulation of inflammatory response to antigenic stimulus, and acute inflammatory response (**Supplemental Table 2**). As highlighted in the pathway enrichment determination, a total of 81 KEGG signaling pathways were identified accordingly *via P*-adjust <0.05. The computational data principally included pathogenic *Escherichia coli* infection, Human T-cell leukemia virus 1 infection, Human immunodeficiency virus 1 infection, Human papillomavirus infection, Kaposi sarcoma-associated herpesvirus infection, Epstein-Barr virus infection, Human cytomegalovirus infection, Endocrine resistance, Progesterone-mediated oocyte maturation, T cell receptor signaling pathway, B cell receptor signaling pathway, Th1 and Th2 cell differentiation, TGF-beta signaling pathway, Natural killer cell-mediated cytotoxicity, IL-17 signaling pathway, Th17 cell differentiation, Inflammatory bowel disease, NF-kappa B signaling pathway, Toll-like receptor signaling pathway, and TNF signaling pathway (**Supplemental Table 3** **)**.

### Protein-Protein Interaction Network, Core Targets of PLB in the Treatment of COVID-19/UCEC

**Figure 5:**
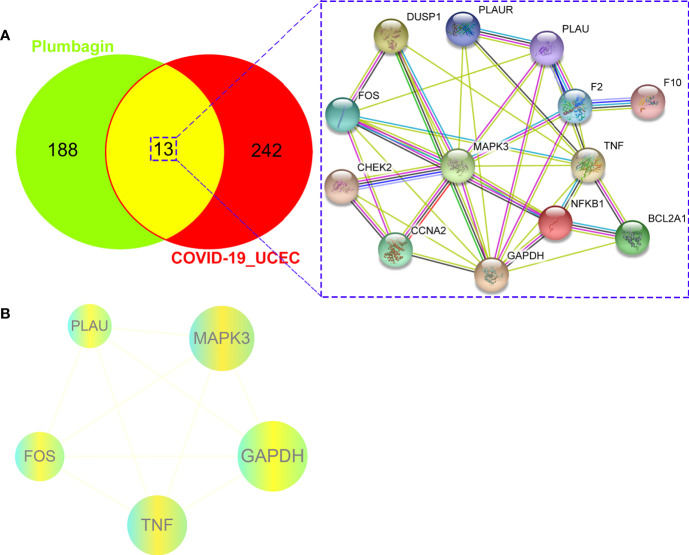
Bioinformatics characteristics of PLB against UCEC/COVID-19. **(A),** a gene-assayed network connection of PLB against UCEC/COVID-19. **(B)**, 5 identified predictive core bio targets of PLB in the potential treatment of UCEC/COVID-19.

We identified a PPI network of PLB in the treatment of COVID-19/UCEC from 13 mutual targets through the STRING database, as shown in **Figure 5A**. Further, all mutual genes were submitted to the Cytoscape 3.7.1 software to determine the topological parameters of the PPI network related to the core targets of PLB in the treatment of COVID-19/UCEC. As a result, we screened and identified 5 final core targets, namely *GAPDH, MAPK3, TNF, FOS*, and *PLAU* (**Figure 5B**).

### Integrative Network Connection

**Figure 6:**
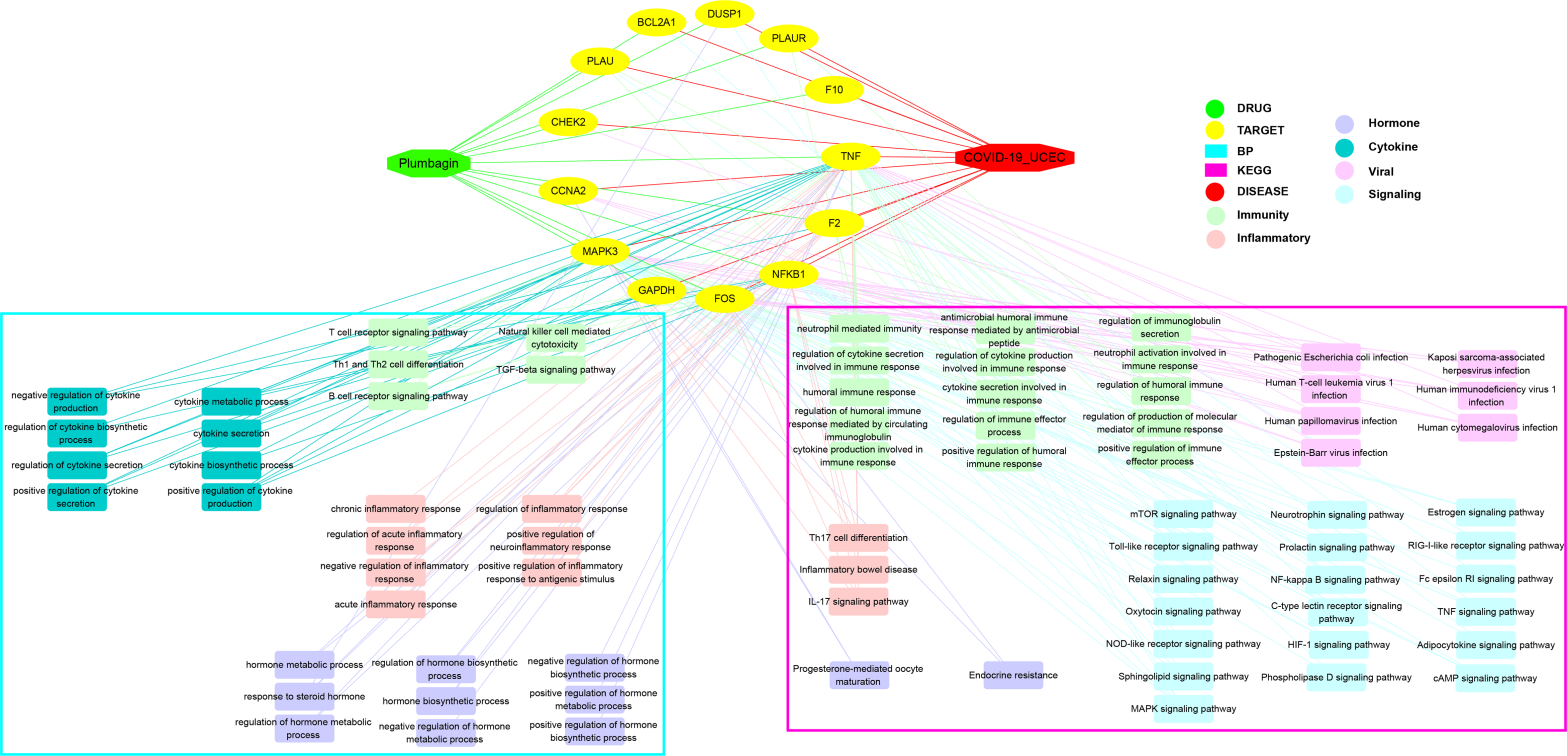
Interaction network connection of candidates, core bio targets, BP, functions, and molecular pathways of PLB in the treatment of UCEC/COVID-19 by using Cytoscape analysis.

Collectively, we used the Cytoscape 3.7.1 software to integrate the bioinformatic findings. As a result, a network connection of PLB-target-BP-KEGG-COVID-19/UCEC was optimized (**Figure 6**).

### Biological Molecular Docking Findings

**Figure 7:**
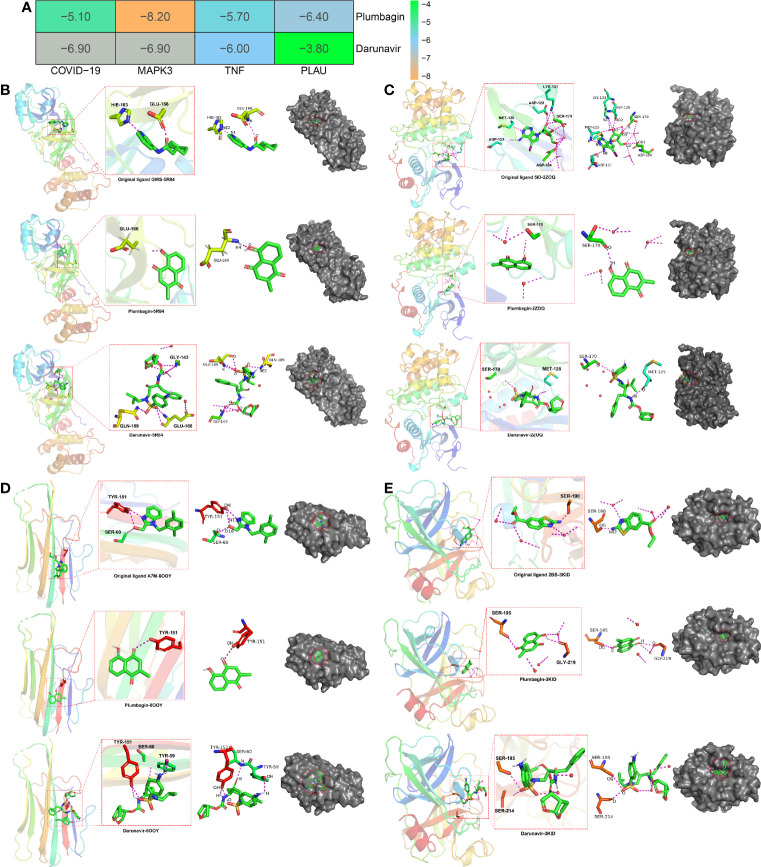
Biological molecular docking findings of PLB against UCEC/COVID-19. **(A)**, molecular affinity and the binding energy of PLB docked with core proteins, including MAPK3, TNF, and PLAU. **(B–E)**, *in silico* characteristics of PLB, docked with core proteins.

Using bioinformatics and computational biology, we determined the active cavities and binding affinity in MAPK3, TNF, and PLAU docked with PLB using the pymol 2.3 software, more docking parameter detail showed in Supplemental material (**Supplemental Tables 4–7**) (**Figure 7A**). In COVID-19 (PDB ID: 5R84) (http://www.rcsb.org/structure/5R84), the root mean squared error (RMSD) of the original ligand GWS was 0.593 Å, and its hydrogen bond with the 5R84 protein acted on the protonation state of HIS-163 -HIE-163 (2.7 Å), and the amino acid residue GLU-166 (3.0 Å). PLB formed a hydrogen bond with the amino acid residue GLU-166 (2.1 Å), and in the surface model, PLB occupied the same active cavity as the original ligand, indicating that it exerted better binding characteristics with the 5R84 protein. Darunavir formed a hydrogen bond with amino acid residues GLY-143 (2.8 Å), GLU-166 (2.8 Å), GLN-189 (2.9 Å) (**Figure 7B**). In MAPK3 (PDB ID: 2ZOQ), the RMSD of the original ligand 5ID was 1.421 Å, which hydrogen-bonded with the ZQOQ protein to act on the amino acid residues ASP-123 (2.6), MET-125 (2.9), ASP- 128 (2.9), LYS-131 (3.3), SER-170 (3.0), and ASP-184 (3.2). PLB formed a hydrogen bond with amino acid residue SER-170 (2.2). In the surface model, plumbagin occupied the same active cavity as the original ligand, and the affinity between PLB and MAPK3 was small, indicating that PLB and the 2ZOQ protein had good binding properties. Darunavir forms a hydrogen bond with amino acid residues MET-125 (2.2 Å) and SER-170 (3.3 Å) (**Figure 7C**). In TNF (PDB ID: 6OOY), the RMSD of the original ligand A7M was 2.980 Å, and its hydrogen bond with the 6OOY protein acted on amino acid residues SER-60 (2.9) and TYR-151 (2.8). PLB formed a hydrogen bond with the amino acid residue TYR-151 (2.8), and in the surface model, PLB occupied the same active cavity as the original ligand, indicating that PLB played better binding characteristics with the 6OOY protein. Darunavir formed a hydrogen bond with amino acid residues TYR-59 (2.5 Å), SER-60 (3.1 Å), TYR-151 (2.5 Å) (**Figure 7D**). In PLAU (PDB ID: 3KID) (http://www.rcsb.org/structure/3KID), the RMSD of the original ligand 2BS was 1.730 Å, and its hydrogen bond with the 3KID protein acted on the amino acid residue SER-190 (2.3). PLB formed hydrogen bonds with amino acid residues SER-195 (2.7) and GLY-219 (2.2). In the surface model, PLB occupied the same active cavity as the original ligand, and the affinity of PLB to PLAU was smaller, indicating that PLB and the 3KID protein had better binding characteristics. Darunavir forms a hydrogen bond with amino acid residues TYR-59 (2.5 Å), SER-195 (3.0 Å), SER-214 (2.5 Å) (**Figure 7E**). In two-dimensional model, more detailed data were presented in **Supplemental Figure 1**.

## Discussion

COVID-19, induced by the newly evolved coronavirus, becomes a cosmopolitical challenge due to SARS-CoV-2 severely threatening human life in many countries (33). Globally, SARS-CoV-2 is evolving and transmitting widespread, leaving a continual increment of death toll, as the specific medicine is absent (34). Fortunately, great efforts are being made to develop a targeting vaccine to suppress SARS-CoV-2 in some countries. However, there is an unknown period of time before the new specific vaccine is completed (35). Statistically, a growing number of people are living with chronic diseases in modern life, such as cardiovascular disorders and cancer. Also, the incidence of these diseases is mounting yearly in the world, especially in western countries (36). In addition, patients suffering from cancer may suffer immunological suppression and dysfunction of immunity, being potentiality prone to hospital-acquired infection (37). In the current grim situation, as in the global outbreak of COVID-19, there is no effective management for handling this problem, producing an increment of the death toll (38). Accordingly, during the early outbreak of COVID-19, hospital-originated infections of this virus were high as it was potentially undetected (2). Mounting epidemiological evidence shows that the cases of UCEC, characterized by malignant metastasis, are increasing around the world (39). As a result, numerous hospitalized cancer patients might be at an increased risk of infection with the new coronavirus, especially during the early outbreak. More significantly, the current treatment against UCEC shows reduced pharmacological effectiveness when over infection with SARS-CoV-2, causing unwanted increment in mortality.

Reportedly, PLB, a naturally occurring naphthoquinone, has been found to possess pharmaceutical anticancer properties *via* cytotoxic action against human cancer cell lines (11). In preliminary mechanism studies, the anti-cervical carcinoma activity of PLB was linked to the induction of apoptosis. In addition, based on the marked anti-inflammatory benefits of PLB, we preliminarily hypothesized that PLB may have effective pharmacological activities in patients with CC/COVID-19. Following an *in silico* investigation, all candidates, 255 mutual and 13 specific genes of COVID-19/UCEC, were screened out. The DGE determination resulted in 108 up-regulated and 147 down-regulated genes in COVID-19/UCEC patients. Accordingly, these DGE-assayed findings are likely to characterize the UCEC cases infected with SARS-CoV-2. In further independent prognostic and survival assays, some of the key differentially expressed genes, such as CCL2, ANPEP, CLEC4M, SCARA3, CP, ABCA4, KHK, SLC8A1, ZYG11B, GHR, TNF, FOSL2, and PLAU, may be used as molecular markers to detect and identify and stage UCEC patients infected with the novel coronavirus. Overall, current clinical investigations demonstrate that 255 mutual genes in COVID-19/UCEC patients are likely to function as candidate therapeutical bio targets. By the use of network pharmacology analysis, we screened out and determined a total of 13 mutual genes with PLB in the treatment COVID-19/UCEC before core target identification. Further analysis identified 5 core targets, including GAPDH, MAPK3, TNF, FOS, and PLAU. The current evidence highlighted that the core genes may be the pharmacologically active bio targets of PLB in the treatment of COVID-19/UCEC. Following an integrative enrichment assay, the bioinformatic data revealed that the anti-COVID-19/UCEC function and mechanism mediated by PLB might effectively be achieved by cytotoxicity, anti-proliferation, inducing apoptosis, anti-inflammation, immunomodulation, and modulation of some of key molecular pathways, such as Human T-cell leukemia virus 1 infection, Human immunodeficiency virus 1 infection, Human cytomegalovirus infection, T cell receptor signaling pathway, B cell receptor signaling pathway, Th1 and Th2 cell differentiation, TGF-beta signaling pathway, Natural killer cell-mediated cytotoxicity, IL-17 signaling pathway, Th17 cell differentiation, NF-kappa B signaling pathway, Toll-like receptor signaling pathway, and TNF signaling pathway. Based on the biological molecular docking method, the anti-COVID-19/UCEC effect of PLB could be achieved by some of the core genes, including MAPK3, TNF, and PLAU, as PLB exerted better active cavities and binding affinity in MAPK3, TNF, and PLAU when docking. In view of these results, we hypothesize that PLB is a likely candidate to be used for the potential treatment of UCEC patients infected with SARS-CoV-2 in the current evolving situation before future clinical trials.

## Conclusions

The current bioinformatic and computational findings reveal the anti-COVID-19/UCEC pharmacological functions and mechanisms achieved by PLB. Moreover, all core targets of PLB treatment in COVID-19/UCEC were identified, indicating potential pharmacological significance. Interestingly, biological molecular docking data indicate that PLB is a likely candidate to be applied clinically in the therapy of UCEC patients infected with SARS-CoV-2.

## Data Availability Statement

The original contributions presented in the study are included in the article/**Supplementary Material**. Further inquiries can be directed to the corresponding authors.

## Author Contributions

RL and MS conceived and designed the study. YML and SY performed the data analysis and data interpretation. YML and XL conducted the bioinformatics and statistical analyses. RL, YML, and SY prepared the manuscript. All authors contributed to the article and approved the submitted version.

## Funding

This study is supported by the National Natural Science Foundation of China (No. 81660091) and the National Natural Science Foundation of Guangxi (No. 2019GXNSFBA185015, 2018GXNSFAA281242).

## Conflict of Interest

The authors declare that the research was conducted in the absence of any commercial or financial relationships that could be construed as a potential conflict of interest.

## Publisher’s Note

All claims expressed in this article are solely those of the authors and do not necessarily represent those of their affiliated organizations, or those of the publisher, the editors and the reviewers. Any product that may be evaluated in this article, or claim that may be made by its manufacturer, is not guaranteed or endorsed by the publisher.

## Abbreviations

UCEC, uterine corpus endometrial carcinoma; COVID-19, coronavirus disease 2019; SARS-CoV-2, severe acute respiratory syndrome coronavirus 2; TCGA, The Cancer Genome Atlas; GO, Gene Ontology; BP, Biological process; KEGG, Kyoto Encyclopedia of Genes and Genomes; RMSD, root mean square deviation; TCMSP, Traditional Chinese Medicine Systems Pharmacology Database and Analysis Platform. Plumbagin, PLB; GEQ, Expression Quantification; FDR, false discovery rate; OMIM, Online Mendelian Inheritance in Man; TTD, Therapeutic Target database; NCBI, National Center for Biotechnology Information; PPI, Protein-protein interaction.

## References

[1] Kakar A Nundy S. COVID-19 in India. J R Soc Med (2020) 113(6):232–3. doi: 10.1177/0141076820927668 PMC7439587 32521202

[2] Moghadas SM Shoukat A Fitzpatrick MC Wells CR Sah P Pandey A. Projecting Hospital Utilization During the COVID-19 Outbreaks in the United States. Proc Natl Acad Sci USA (2020) 117:9122–6. doi: 10.1073/pnas.2004064117 PMC7183199 32245814

[3] Esposito S Noviello S Pagliano P. Update on Treatment of COVID-19: Ongoing Studies Between Promising and Disappointing Results. Infez Med (2020) 28:198–211. 32335561

[4] Russell CD Millar JE Baillie JK. Clinical Evidence Does Not Support Corticosteroid Treatment for 2019-Ncov Lung Injury. Lancet (2020) 395:473–5. doi: 10.1016/S0140-6736(20)30317-2 PMC7134694 32043983

[5] Harky A Chiu CM Yau THL Lai SHD. Cancer Patient Care During COVID-19. Cancer Cell (2020) 37:749–50. doi: 10.1016/j.ccell.2020.05.006 PMC7221386 32410898

[6] MacKintosh ML Crosbie EJ. Prevention Strategies in Endometrial Carcinoma. Curr Oncol Rep (2018) 20:101. doi: 10.1007/s11912-018-0747-1 30426278 PMC6244901

[7] Amant F Moerman P Neven P Timmerman D Van Limbergen E Vergote I. Endometrial Cancer. Lancet (2005) 366:491–505. doi: 10.1016/S0140-6736(05)67063-8 16084259

[8] Zhou JY Zhang L Wei LH Wang JL. Endometrial Carcinoma-Related Genetic Factors: Application to Research and Clinical Practice in China. BJOG (2016) 123:90–6. doi: 10.1111/1471-0528.14007 27627606

[9] Wee LE Conceicao EP Sim XYJ Aung MK Tan KY Wong HM. Minimizing Intra-Hospital Transmission of COVID-19: The Role of Social Distancing. J Hosp Infect (2020) 105:113–5. doi: 10.1016/j.jhin.2020.04.016 PMC7152925 32294511

[10] Pai SA Munshi RP Panchal FH Gaur IS Mestry SN Gursahani MS. Plumbagin Reduces Obesity and Nonalcoholic Fatty Liver Disease Induced by Fructose in Rats Through Regulation of Lipid Metabolism, Inflammation and Oxidative Stress. BioMed Pharmacother (2019) 111:686–94. doi: 10.1016/j.biopha.2018.12.139 30611993

[11] Tripathi SK Panda M Biswal BK. Emerging Role of Plumbagin: Cytotoxic Potential and Pharmaceutical Relevance Towards Cancer Therapy. Food Chem Toxicol (2019) 125:566–82. doi: 10.1016/j.fct.2019.01.018 30685472

[12] Sakunrangsit N Ketchart W. Plumbagin Inhibited AKT Signaling Pathway in HER-2 Overexpressed-Endocrine Resistant Breast Cancer Cells. Eur J Pharmacol (2020) 868:172878. doi: 10.1016/j.ejphar.2019.172878 31863768

[13] Sakunrangsit N Ketchart W. Plumbagin Inhibits Cancer Stem-Like Cells, Angiogenesis and Suppresses Cell Proliferation and Invasion by Targeting Wnt/β-Catenin Pathway in Endocrine Resistant Breast Cancer. Pharmacol Res (2019) 150:104517. doi: 10.1016/j.phrs.2019.104517 31693936

[14] Pan Q Zhou R Su M Li R. The Effects of Plumbagin on Pancreatic Cancer: A Mechanistic Network Pharmacology Approach. Med Sci Monit (2019) 25:4648–54. doi: 10.12659/MSM.917240 PMC6604675 31230062

[15] Zhou R Wu K Su M Li R. Bioinformatic and Experimental Data Decipher the Pharmacological Targets and Mechanisms of Plumbagin Against Hepatocellular Carcinoma. Environ Toxicol Pharmacol (2019) 70:103200. doi: 10.1016/j.etap.2019.103200 31158732

[16] Srinivas P Gopinath G Banerji A Dinakar A Srinivas G. Plumbagin Induces Reactive Oxygen Species, Which Mediate Apoptosis in Human Cervical Cancer Cells. Mol Carcinog (2004) 40:201–11. doi: 10.1002/mc.20031 15264212

[17] Nair S Nair RR Srinivas P Srinivas G Pillai MR. Radiosensitizing Effects of Plumbagin in Cervical Cancer Cells Is Through Modulation of Apoptotic Pathway. Mol Carcinog (2008) 47:22–33. doi: 10.1002/mc.20359 17562542

[18] Luo P Wong YF Ge L Zhang ZF Liu Y Liu L. Anti-Inflammatory and Analgesic Effect of Plumbagin Through Inhibition of Nuclear Factor-κb Activation. J Pharmacol Exp Ther (2010) 335:735–42. doi: 10.1124/jpet.110.170852 20858709

[19] Bhattacharya A Jindal B Singh P Datta A Panda D. Plumbagin Inhibits Cytokinesis in Bacillus Subtilis by Inhibiting FtsZ Assembly–A Mechanistic Study of Its Antibacterial Activity. FEBS J (2013) 280:4585–99. doi: 10.1111/febs.12429 23841620

[20] Periasamy H Iswarya S Pavithra N Senthilnathan S Gnanamani A. *In Vitro* Antibacterial Activity of Plumbagin Isolated From Plumbago Zeylanica L. Against Methicillin-Resistant Staphylococcus Aureus. Lett Appl Microbiol (2019) 69:41–9. doi: 10.1111/lam.13160 31044446

[21] Wu K Wei P Liu M Liang X Su M. To Reveal Pharmacological Targets and Molecular Mechanisms of Curcumol Against Interstitial Cystitis. J Adv Res (2019) 20:43–50. doi: 10.1016/j.jare.2019.05.003 31193808 PMC6543129

[22] Li R Ma XY Song YQ Zhang YY Xiong WB Li L. Anti-Colorectal Cancer Targets of Resveratrol and Biological Molecular Mechanism: Analyses of Network Pharmacology, Human and Experimental Data. J Cell Biochem (2019) 120:11265–73. doi: 10.1002/jcb.28404 30719773

[23] Li R Guo C Li Y Liang X Yang L Huang W. Therapeutic Target and Molecular Mechanism of Vitamin C-Treated Pneumonia: A Systematic Study of Network Pharmacology. Food Funct (2020) 11:4765–72. doi: 10.1039/D0FO00421A 32420559

[24] Li R Song Y Ji Z Li L Zhou L. Pharmacological Biotargets and the Molecular Mechanisms of Oxyresveratrol Treating Colorectalcancer: Network and Experimental Analyses. Biofactors (2020) 46:158–67. doi: 10.1002/biof.1583 31647596

[25] Liang Y Zhou R Liang X Kong X Yang B. Pharmacological Targets and Molecular Mechanisms of Plumbagin to Treat Colorectal Cancer: A Systematic Pharmacology Study. Eur J Pharmacol (2020) 881:173227. doi: 10.1016/j.ejphar.2020.173227 32505664

[26] Su M Guo C Liu M Liang X Yang B. Therapeutic Targets of Vitamin C on Liver Injury and Associated Biological Mechanisms: A Study of Network Pharmacology. Int Immunopharmacol (2019) 66:383–7. doi: 10.1016/j.intimp.2018.11.048 30530052

[27] Li R Li Y Liang X Yang L Su M Lai KP. Network Pharmacology and Bioinformatics Analyses Identify Intersection Genes of Niacin and COVID-19 as Potential Therapeutic Targets. Brief Bioinform (2021) 22:1279–90. doi: 10.1093/bib/bbaa300 PMC7717147 33169132

[28] Li R Wu K Li Y Liang X Tse WKF Yang L. Revealing the Targets and Mechanisms of Vitamin A in the Treatment of COVID-19. Aging (Albany NY) (2020) 12:15784–96. doi: 10.18632/aging.103888 PMC7467385 32805728

[29] Liu F Pan Q Wang L Yi S Liu P Huang W. Anticancer Targets and Mechanisms of Calycosin to Treat Nasopharyngeal Carcinoma. Biofactors (2020) 46:675–84. doi: 10.1002/biof.1639 32449282

[30] Li R Guo C Li Y Qin ZQ Huang WJ. Therapeutic Targets and Signaling Mechanisms of Vitamin C Activity Against Sepsis: A Bioinformatics Study. Brief Bioinform (2021) 22(3):bbaa079. doi: 10.1093/bib/bbaa079 32393985 PMC7454291

[31] Li R Guo C Li Y Liang X Su M. Functional Benefit and Molecular Mechanism of Vitamin C Against Perfluorooctanesulfonate-Associated Leukemia. Chemosphere (2021) 263:128242. doi: 10.1016/j.chemosphere.2020.128242 33297189

[32] Li R Wu K Li Y Liang X Lai KP Chen J. Integrative Pharmacological Mechanism of Vitamin C Combined With Glycyrrhizic Acid Against COVID-19: Findings of Bioinformatics Analyses. Brief Bioinform (2021) 22:1161–74. doi: 10.1093/bib/bbaa141 PMC7462346 32662814

[33] Lai CC Shih TP Ko WC Tang HJ Hsueh PR. Severe Acute Respiratory Syndrome Coronavirus 2 (SARS-CoV-2) and Coronavirus Disease-2019 (COVID-19): The Epidemic and the Challenges. Int J Antimicrob Agents (2020) 55:105924. doi: 10.1016/j.ijantimicag.2020.105924 32081636 PMC7127800

[34] McKee DL Sternberg A Stange U Laufer S Naujokat C. Candidate Drugs Against SARS-CoV-2 and COVID-19. Pharmacol Res (2020) 157:104859. doi: 10.1016/j.phrs.2020.104859 32360480 PMC7189851

[35] Dror AA Eisenbach N Taiber S Morozov NG Mizrachi M Zigron A. Vaccine Hesitancy: The Next Challenge in the Fight Against COVID-19. Eur J Epidemiol (2020) 35:775–9. doi: 10.1007/s10654-020-00671-y PMC8851308 32785815

[36] Cockerham WC Hamby BW Oates GR. The Social Determinants of Chronic Disease. Am J Prev Med (2017) 52:5–12. doi: 10.1016/j.amepre.2016.09.010 PMC5328595 27989293

[37] Ariza-Heredia EJ Chemaly RF. Update on Infection Control Practices in Cancer Hospitals. CA Cancer J Clin (2018) 68:340–55. doi: 10.3322/caac.21462 PMC7162018 29985544

[38] Weinberger DM Cohen T Crawford FW Mostashari F Olson D Pitzer VE. Estimating the Early Death Toll of COVID-19 in the United States. bioRxiv (2020). doi: 10.1101/2020.04.15.20066431

[39] Sorosky JI. Endometrial Cancer. Obstet Gynecol (2012) 120:383–97. doi: 10.1097/AOG.0b013e3182605bf1 22825101

