# Supplementary figures and images for: Medical Significance of Uterine Corpus Endometrial Carcinoma Patients Infected With SARS-CoV-2 and Pharmacological Characteristics of Plumbagin
Source: Front Endocrinol (Lausanne). 2021 Oct 12;12:714909. doi: 10.3389/fendo.2021.714909 (PMC8547653; doi:10.3389/fendo.2021.714909)

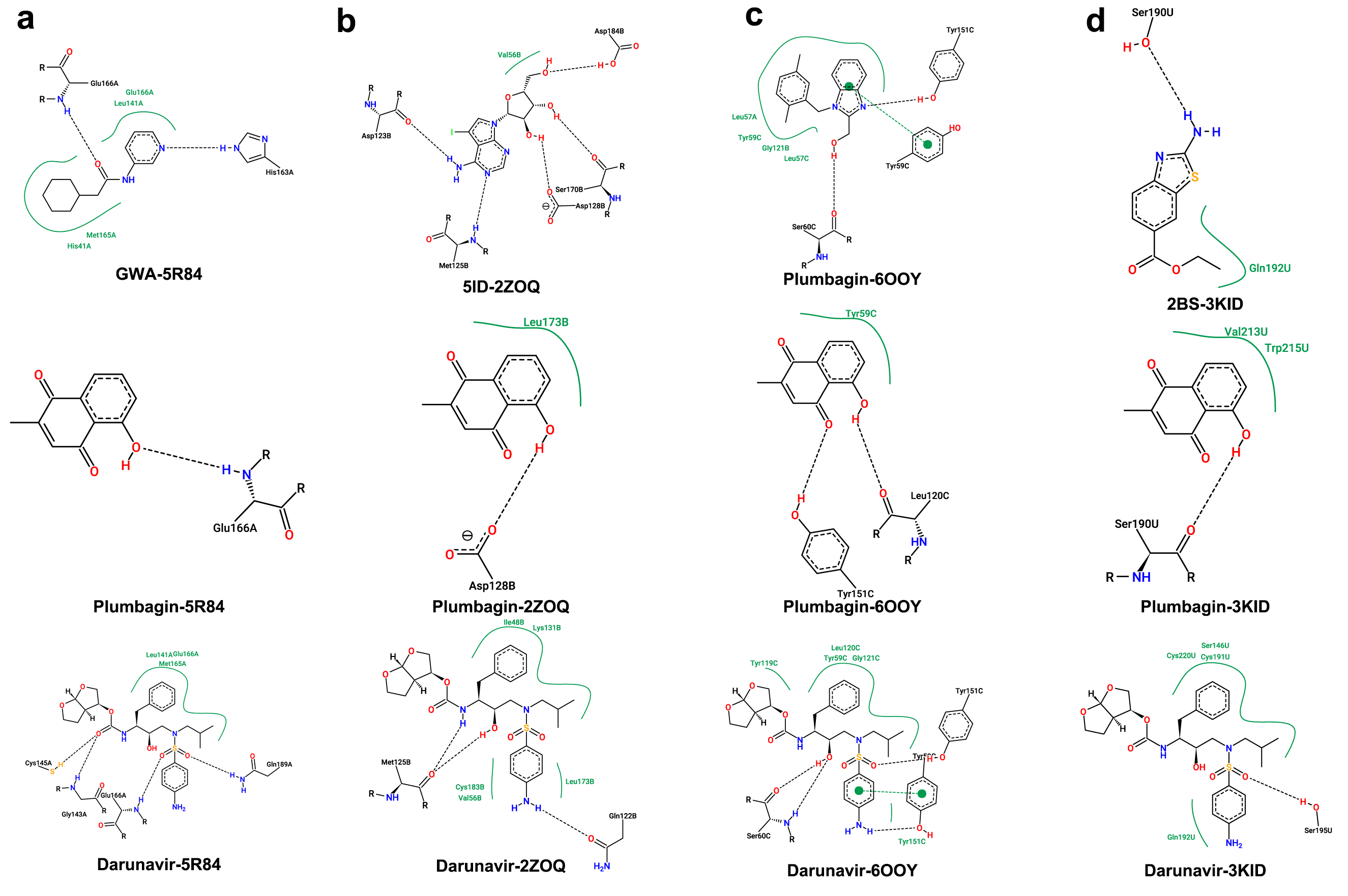

Supplement: Supplementary file 1 [file Image_1.tif]
