# Supplementary material for: Medical Significance of Uterine Corpus Endometrial Carcinoma Patients Infected With SARS-CoV-2 and Pharmacological Characteristics of Plumbagin
Source: Front Endocrinol (Lausanne). 2021 Oct 12;12:714909. doi: 10.3389/fendo.2021.714909 (PMC8547653; doi:10.3389/fendo.2021.714909)
Supplement: Supplementary file 8 [file Table_7.docx]

**Supplemental Table 7**.

The docking parameter of PLAU (PDB ID: 3KID)

| Docking parameter of PLAU (PDB ID: 3KID) | | |
| --- | --- | --- |
| Exhaustiveness arg | | 8 (Default) |
| Grid Zize | Number of point in x-dimension | 30 |
|  | Number of point in y-dimension | 30 |
|  | Number of point in z-dimension | 30 |
|  | X center | -0.917 |
|  | Y center | -32.595 |
|  | Z center | -11.125 |
| Spacing (angstrom) | | 0.375 |
| Number of modes | | 9 (default) |
